# An evolving role in the NICU: a study of the tasks, knowledge, skills, and practice demographics of the neonatal therapist

**DOI:** 10.3389/fped.2025.1677432

**Published:** 2025-11-26

**Authors:** Alicia Fernandez-Fernandez, Deanna Gibbs, Margaret Miller, Kati Knudsen, Louisa Ferrara-Gonzalez, Roberta Pineda

**Affiliations:** 1 Physical Therapy Department, School of Rehabilitative Sciences, Dr. Kiran C. Patel College of Osteopathic Medicine, Nova Southeastern University, Fort Lauderdale, FL, United States; 2 Neonatal Intensive Care Unit, South Miami Hospital, Miami, FL, United States; 3 Corporate Nursing Directorate, Barts Health NHS Trust, London, United Kingdom; 4 Providence Sacred Heart Medical Center, Spokane, WA, United States; 5 Neonatal Intensive Care Unit, Providence St. Vincent Medical Center, Portland, OR, United States; 6 Department of Pediatrics, NYU Langone Hospital—Long Island, Mineola, NY, United States; 7 Infant Feeding Specialists, Inc., Garden City, NY, United States; 8 Chan Division of Occupational Science and Occupational Therapy, University of Southern California, Los Angeles, CA, United States; 9 Department of Pediatrics, Keck School of Medicine, Los Angeles, CA, United States; 10 Gehr Family Center for Health Systems Science and Innovation, University of Southern California, Los Angeles, CA, United States; 11 Program in Occupational Therapy, Washington University, St. Louis, MO, United States

**Keywords:** neonatal therapy, occupational therapy, physical therapy, speech-languagepathology, certification, practice, roles, practice analysis

## Abstract

**Objectives:**

To describe current practice and roles of the neonatal therapist.

**Study design:**

A Neonatal Therapy Practice Analysis was distributed to neonatal therapists (physical therapists, occupational therapists, and speech-language pathologists) in 2019–2020 via social media, email, newsletters, and conference materials.

**Results:**

There were 1,313 respondents from 1,110 different hospitals. 41.7% (*n* = 277) were occupational therapists, 29.3% (*n* = 195) were physical therapists, and 29.0% (*n* = 193) were speech-language pathologists. 760 (59.1%) worked in level III NICUs, while 248 (19.3%) worked in level II NICUs and 273 (21.2%) in level IV NICUs. 166 (28.1%) of respondents were Certified Neonatal Therapists (CNTs), which was related to higher number of full-time equivalent (FTE) positions per bed (*β* = 1.066, SE = 0.478, *p* = 0.026) and higher percentage of infants served in the NICU (*β* = −3.2, SE = 0.589, *p* < 0.001). We observed a median of one therapy FTE per 17 NICU beds (range of 1 FTE per 10–200 beds). Higher acuity NICU levels (*β* = 2.23, SE = 0.197, *p* < 0.001) and NICUs with higher number of beds (*β* = 2.497, SE = 0.285, *p* < 0.001) had more neonatal therapy FTEs. Survey respondents reported working with a median of 76.0% of infants in their respective NICUs (IQR 65%–90%, range 1%–100%). There was a higher percentage of infants served in higher acuity NICUs (*β* = 4.358, SE = 1.517, *p* = 0.004), in NICUs with a higher number of beds (*β* = 0.058, SE = 0.029, *p* = 0.047), when there was a productivity standard (*β*=11.47, SE = 1.9, *p* < 0.001), and where there was a higher number of neonatal therapy FTEs (*β* = 1.0, SE = 0.239, *p* < 0.001). 294 (46%) of respondents reported having standing orders, which was related to a higher percentage of infants served in the NICU (*β* = −1.109, SE = 0.393, *p* < 0.001) and to having a productivity standard (*β* = −0.467, SE = 0.139, *p* < 0.001). 65.3% (*n* = 415) of respondents reported having productivity standards to meet each day, with a range between 50%–80%.

**Conclusion:**

This practice analysis provides insights into the changing landscape of neonatal therapy.

## 1 Introduction

Neonatal therapy is recognized as an advanced area of practice by the major therapy organizations in the United States (US) including the American Physical Therapy Association (APTA), the American Occupational Therapy Association (AOTA), and the American Speech and Hearing Association (ASHA) (1–3). The American Academy of Pediatrics (AAP) also recommends neonatal therapists be available to support development and feeding in level II, III and IV neonatal intensive care units (NICUs) (4, 5). Further, guidelines or position statements related to recommended staffing of neonatal therapists have been disseminated (6).

There is a growing body of evidence identifying neonatal therapy practice. In 2013, Sturdivant published survey results from 238 neonatal therapists as she sought to define who a neonatal therapist is, along with components of neonatal therapy practice. She identified that neonatal therapists have a significant amount of clinical experience within this specialized practice setting, and recognized that neonatal therapists used a variety of treatment models in practice (7). In 2017, Ross and colleagues identified the presence of a multidisciplinary team within a large, single level IV NICU as well as defined referral and intervention patterns (8). In 2020, perceptions about the roles of neonatal therapists, as well as recommendations for neonatal therapy staffing in the NICU, were published (6). In addition to these reports, the Neonatal Therapy Certification Board (NTCB®) has taken an active role in defining the neonatal therapy landscape and current practice since its inception in 2014.

It is important for a certifying body to clearly understand the comprehensive tasks that a given profession may conduct, so that the certification exam can closely align with current practice (9). Further, within a rapidly changing field, updated practice analyses should inform future generations of the exam across time. The first NTCB practice analysis was conducted between 2015 and 2016 and published in 2019 (10). There were 468 neonatal therapists who participated in the first practice analysis of which 208 (47%) were occupational therapists (OTs), 140 (32%) were physical therapists (PTs), and 94 (21%) were speech-language pathologists (SLPs). Half of respondents had a doctorate degree, and a large number reported practicing for >5 years before entering practice in the NICU. A very high proportion of respondents believed that oversight in the NICU is critical, and almost all respondents were interested in a neonatal therapy certification program to establish valid criteria for practicing in this advanced area of practice. This initial practice analysis also defined some key components of NICU-based therapy and set the stage for developing the Neonatal Therapy Certification® process and Neonatal Therapy Certification Exam®.

Neonatal therapy is a rapidly growing field as there is increased awareness of and attention to the developmental risks for infants in the NICU, as well as enhanced focus on modifiable neuroprotective strategies that can be used to optimize outcomes. Therefore, practice analyses completed at routine intervals are necessary to capture these changes in clinical practice and to ensure the certification exam reflects current practice. The NTCB compiles and employs routine practice analyses to inform the ongoing work of certification, by conducting a practice analysis at least every 5 years and a comprehensive practice analysis at least every 10 years. In 2019–2020, another practice analysis was launched to systematically understand the tasks that neonatal therapists conduct and what knowledge is needed to conduct them. This practice analysis led to the launch of the 2nd generation of the Neonatal Therapy Certification Exam. From this data, we have reported on current neonatal therapy staffing in US-based NICUs and estimated there were 4,232 neonatal therapists in the US, with most hospitals having a dedicated team of neonatal therapists for the NICU. Higher acuity level IV NICUs were more likely to have a dedicated team than lower acuity NICUs. At that time (2–3 years after Neonatal Therapy Certification was launched; in 2020), there were more than 300 Certified Neonatal Therapists (CNTs) with 22% of NICUs having at least one CNT (11).

While the first practice analysis in 2015–2016 largely aided our understanding of the need for a certification process (10), in addition to defining parameters of current practice, the 2019–2020 practice analysis focused attention on the role of neonatal therapy. It specifically aided in comprehensively defining the role of the neonatal therapist, the knowledge needed to complete that role, different patterns of neonatal therapy delivery, and key trends across neonatal therapy practice (11).

## 2 Methods

*Neonatal Therapy Practice Analysis* (2019–2020)*.* This practice analysis was designed by Dr. Roberta Pineda, Dr. Deanna Gibbs, and Dr. Alicia Fernandez-Fernandez with input from several other members of the NTCB. The 2019–2020 practice analysis contained approximately 60 questions, focused on obtaining specific neonatal therapy practice demographics, and took participants approximately 30 min to complete. An additional portion of the survey (the last set of questions) was only for Certified Neonatal Therapists and included 44 constructs examining the knowledge, scope, and practices of neonatal therapists (OT, PT & SLP) to enable an understanding of critical components of knowledge in neonatal therapy practice, and how often they are utilized. These additional findings will be analyzed and reported separately.

*Survey Development and Piloting*. The questions on the practice analysis were conceptualized and went through an iterative process of review by the practice analysis committee to aid clarity of each question until there was consensus on inclusion and wording. The survey was then piloted on members of the NTCB and members of the National Association of Neonatal Therapists (NANT) who not only answered the survey questions but also provided feedback on questions requiring modification. This process continued with members of the NTCB until no additional feedback was provided. After a final review by the practice analysis committee, the survey was finalized and loaded into Research Electronic Data Capture (REDCap). See supplementary material for survey questions asked of neonatal therapist respondents that are relevant to this manuscript. This survey-based study was approved by the Washington University Human Research Protection Office.

*Participants.* (1) Inclusion criteria: The National Association of Neonatal Therapists (NANT) defines a neonatal therapist as an occupational therapist, physical therapist, or speech-language pathologist. Thus, neonatal therapists (OTs, PTs, and SLPs) who had practiced or were practicing on a full-time, part-time, or consultative basis in a Level II, III, or IV NICU were invited to participate in the survey. All neonatal OTs, PTs, and SLPs in the United States, as well as in international locations, were included. (2) Exclusion criteria: Occupational therapists, physical therapists, or speech-language pathologists who had not practiced in a NICU were excluded. All other disciplines that may work in the NICU (such as music therapists, nursing, neonatologists, psychologists) were excluded. These disciplines do not fit the definition of neonatal therapist and have different levels of education and training.

*Recruitment.* Invitations to participate in the web-based survey in REDCap were disseminated with a link via email (from neonatal practitioners or from neonatal therapists to known colleagues), through a post in a weekly newsletter by NANT to its active email list, through a newsletter by the NTCB, through social media platforms visited by neonatal therapists, through requests via postcard mailing and phone calls to hospitals contained in national databases, and via conference materials. The number of neonatal therapists reached is unknown, therefore the response rate cannot be calculated. However, we are able to estimate the response rate based on our previous neonatal therapy staffing estimates (11). Respondents were free to skip any of the questions on the survey, and all data that was collected from each respondent was used in analyses. Due to data collection methods, there was no direct way to verify that a therapist only took the survey once.

*Statistical analysis.* Planned descriptive statistics included counts and frequencies of responses for categorical variables, means and standard deviations for normally-distributed continuous variables, and medians and IQRs for non-normally-distributed continuous variables. Because none of the continuous variables explored in this manuscript were normally distributed, medians and IQRs were reported in that case. Range was also reported for variables where it could be informative to describe the scope of variation across responses. ChatGPT was used to enrich the organization of qualitative responses with free text fields in conjunction with human supervision and control to ensure accuracy and depth of analysis, as advised by Torobov (12, 13). To define the top ten job-related activities in which neonatal therapists participate, the percentage of therapists who reported they conducted each identified task was defined, followed by determining the ten activities with the highest percentages. The same process was used to determine the top ten assessment tools neonatal therapists use in the NICU. In addition, we investigated:

- Whether productivity standards were related to NICU acuity level, number of beds, discipline, percentage of NICU time, and who the therapist reports to (directly to NICU vs. rehabilitation or other departments), using chi-square analyses and logistic regression.
- Whether the number of neonatal therapy FTEs were related to NICU acuity level and number of beds using linear regression models. We also investigated the number of FTEs per bed and its relationship with number of CNTs and percentage of infants served by therapy in the NICU using linear regression.
- The relationships between having standing orders and level of NICU, number of NICU beds, who the therapist reports to, percentage of infants served in the NICU, number of FTEs, and whether there is a productivity standard using logistic regression models.
- Whether the reported percentage of infants served in the NICU was related to NICU level, number of beds, number of FTEs, having productivity standards, whether the therapist reports directly to the NICU, and whether the therapist is a CNT was investigated using linear regression models.

## 3 Results

**Figure 1:**
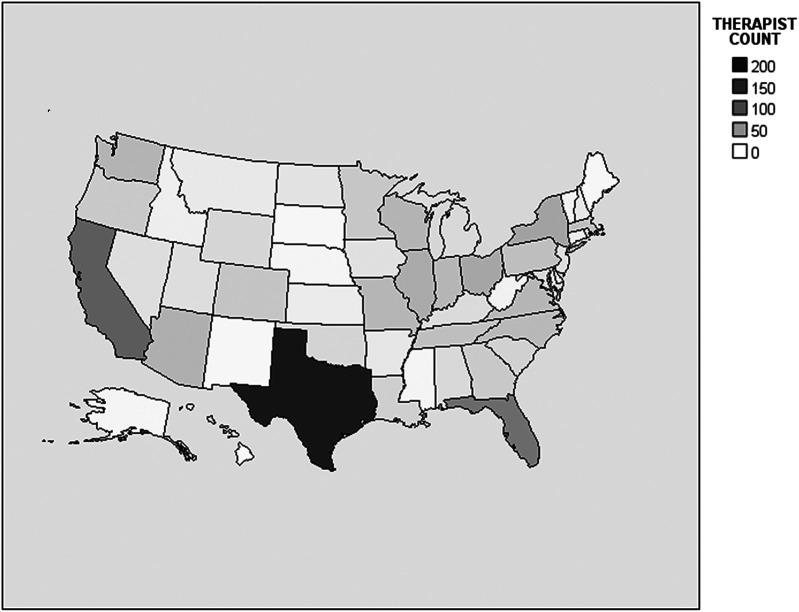
Distribution of neonatal therapy respondents across different US states. Color scale legend indicates minimum number associated with a given color. For instance, 0 = 0 to 49, 50 = 50 to 99, etc.

**Figure 2:**
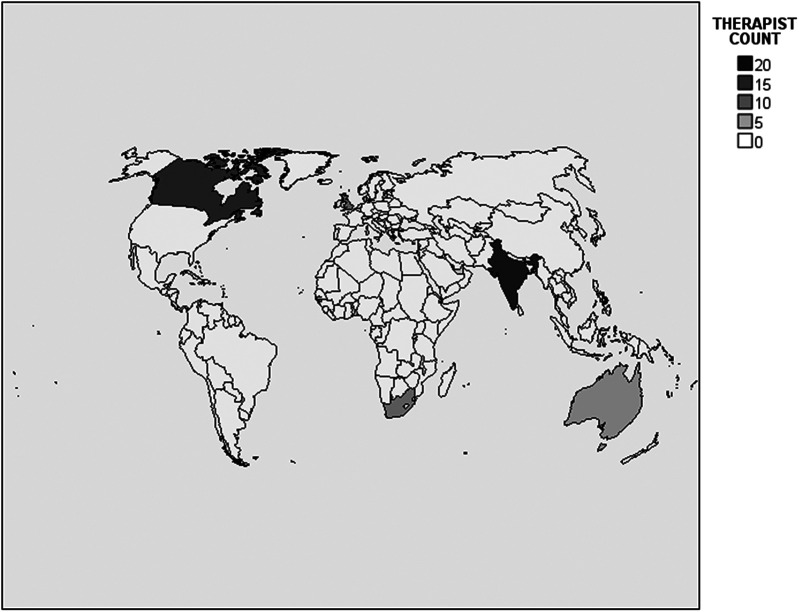
Distribution of neonatal therapy survey respondents across different countries. The US is excluded from color coding in this map to avoid scale distortion, as over 97% of respondents were in the US. Color scale legend indicates minimum number associated with a given color. For instance, 0 = 0 to 4, 5 = 5 to 9, etc.

The practice analysis had 1,313 respondents from 1,110 different hospitals. All results reported below are calculated based on available responses for each variable. Representation was included from all US states, including Washington DC as well as the US territory of Puerto Rico, and 34 (2.6%) of the respondents were located outside of the US. See Figures 1, 2 for the distribution of neonatal therapy survey respondents across different US states and countries. All respondents were neonatal therapists, and out of 665 respondents who identified their discipline, there were 277 (41.7%) OTs, 195 (29.3%) PTs, and 193 (29.0%) SLPs.

### 3.1 Neonatal therapist characteristics

The neonatal therapists surveyed had 16.5 median total years of clinical experience (IQR 10.1–23.0 years, range 1–40 years), 5,200 median total hours of NICU experience (IQR 2,100–10,000 h; range 100–95,000 h), 40.0 median work hours per week (IQR 32.0–40.0 h, range 2 to 60 h) and 25.0 median NICU work hours per week (IQR 15–36 h, range 0.5 to 60 h), and 82 (12.8%) of respondents reported working at more than one hospital.

Not all neonatal therapists were dedicated to the NICU, and some practiced and/or served in other departments or areas in a hospital or within health care. Therapists also had different reporting patterns. Table 1 shows information on the practice environment of neonatal therapists, including the departments and areas they serve and support, as well as their reporting departments. Totals exceed 100% for department affiliation and areas supported, as respondents may report to multiple departments and work in several areas in addition to the NICU.

**Table 1:** Practice environment of neonatal therapists: work time in NICU, department affiliation, and other supported areas.

Domain	Subcategory	N	%
Work time in NICU	Consult only	16	2.5%
	<10%	20	3.1%
	11%–24%	39	6.0%
	25%–49%	53	8.2%
	50%–75%	113	17.4%
	76%–99%	212	32.7%
	100%	196	30.2%
Department affiliation/reporting	Adult rehabilitation department	288	43.2%
	Pediatric rehabilitation department	187	28.0%
	Report directly to the NICU	157	23.5%
	Department not listed	93	14.0%
Areas supported outside NICU	NICU follow-up clinic	182	27.3%
	Pediatric acute inpatient	178	26.7%
	Pediatric intensive care unit (PICU)	162	24.3%
	Pediatric outpatient	153	22.9%
	Adult inpatient	134	20.1%
	Administration	119	17.8%
	Mother-baby units	118	17.7%
	Other areas not listed	58	8.7%
	Pediatric inpatient rehabilitation	50	7.5%
	Adult outpatient	19	2.8%
	Developmental daycare	7	1.0%
	Schools	3	0.4%
	Skilled nursing	1	0.1%

In terms of mentoring and continuing education, 546 (84.3%) of therapists reported they had received at least 40 h of mentoring in the NICU. Furthermore, respondents reported keeping up with current practice in different ways including reading journals and/or books (615, 92.2%), education within their hospital (387, 58.0%), education outside of their hospital (627, 94.0%), and collaboration with other peers with expertise in neonatal therapy (608, 91.3%). Therapists reported a median of 45.0 h of NICU-related education over the preceding 3 years (IQR 30.0–60.0).

### 3.2 Neonatal therapy certification

The practice analysis results revealed that respondents were either already certified in neonatal therapy (166, 28.1%), were in the process of becoming a CNT (62, 10.5%), or interested in becoming a CNT in the future (62, 10.5%). Of those surveyed, 171 (29.0%) answered that they were interested in pursuing certification but had not yet begun preparations, and 114 (19.3%) replied that they were uncertain about applying for certification. 26 (4.4%) replied they were not interested in becoming certified in neonatal therapy.

### 3.3 Provision of NICU therapy

Respondents reported a variety of job-related activities during the practice of neonatal therapy, including: (1) education of parents, nurses, physicians and other staff; (2) employing developmental care strategies; (3) developing bedside care plans; (4) participating in discharge planning; (5) positioning treatments; (6) contributing to rounds; providing interventions addressing (7) feeding, (8) sensory, and (9) movement; and (10) supporting referrals to other providers as needed. All of these aforementioned activities were determined via the practice analysis to make up the top ten activities conducted by therapists in the NICU. Other reported activities included co-treatments with other NICU therapists, completing standardized assessments for clinical and/or research purposes, providing care according to a preventative model or rehabilitative model, serving as a bridge to community therapy, splinting, completing feeding studies, chest physical therapy (PT), and conducting research. See Table 2.

**Table 2:** Frequency of neonatal therapy tasks respondents indicated performing as part of their role as a neonatal therapist. Ranking is established based on largest percentage of respondents indicating it is an activity they perform as part of their role as a neonatal therapist.

Task that therapist identified being engaged in (*n* = 667)	*N* (%)	Ranking
Parent education	638 (96%)	1
Developmental care	620 (93%)	2
RN education	611 (92%)	3
Discharge planning	579 (87%)	4
Positioning treatment	535 (80%)	5
Engaged in bedside care plan	523 (78%)	6
Contribute to developmental rounds	514 (77%)	7
Attend developmental rounds	480 (72%)	8
Feeding treatment	469 (70%)	9
Physician education	449 (67%)	10
Sensory treatment	424 (64%)	11
Referrals	422 (63%)	12
Prevention model	408 (61%)	13
Movement treatment	389 (58%)	14
Cotreatment	352 (53%)	15
Bridge to community therapy	344 (52%)	16
Splinting	338 (51%)	17
Attend medical rounds	267 (40%)	18
Standardized assessment clinically	257 (39%)	19/20
Clinical leadership meetings	258 (39%)	19/20
Rehabilitation model	228 (34%)	21
Feeding studies	220 (33%)	22
Engages in research activities for others' studies	146 (22%)	23
Engages in research activities for own study	104 (16%)	24
Standardized assessment for research	84 (13%)	25
Chest physiotherapy	19 (3%)	26

The top standardized assessments used by those surveyed included the Test of Infant Motor Performance (TIMP), Prechtl's General Movements Assessment (GMA), the Bayley Scales of Infant and Toddler Development 3rd edition (Bayley-III), the Newborn Behavioral Assessment Scale (NBAS), the Alberta Infant Motor Scale (AIMS), the Dubowitz Examination or Hammersmith Neonatal Neurological Examination (HNNE), the Morgan, the Peabody, Early Feeding Skills (EFS) Assessment), Use of the Newborn Individualized Developmental Care and Assessment Program (NIDCAP) framework, the Infant Positioning Assessment Tool (IPAT), the Neonatal Oral Motor Assessment Scale (NOMAS), the Neonatal Eating Outcome Assessment (NEO), and the Newborn Behavioral Observation Tool (NBO). See Table 3. 285 (44.9%) respondents reported the existence of developmental rounds, with 267 (82.4%) of those therapists reporting participation in developmental rounds and 148 (46.4%) having a leadership role in such rounds. 368 (58.0%) reported the existence of a developmental care committee, with 346 (85.4%) participating and 239 (57.9%) having a leadership role on such a committee. Medical rounds were most commonly reported to occur daily, twice per week, or weekly (with those three choices totaling 87.9% of reported frequencies), and 240 (37.9%) neonatal therapists reported participating in medical rounds. 248 (39.7%) therapists participated in clinical leadership meetings which were most often held monthly.

**Table 3:** Assessments reported by neonatal therapists.

Assessment Tool^a^ (*n* = 200)	*N* (%)	Ranking
Test of Infant Motor Performance (TIMP)	44 (22%)	1
Prechtl's General Movement Assessment (GMA)	41 (21%)	2
Bayley Scales of Infant Development III (Bayley III)	34 (17%)	3
NBAS (Neonatal Behavioral Assessment Scale)	14 (7%)	4
Alberta Infant Motor Scale (AIMS)	13 (7%)	5/6
Hammersmith Neonatal Neurological Assessment/Dubowitz	13 (7%)	5/6
Morgan/Neonatal Neurological Evaluation (NNE)	12	7
Peabody Developmental Motor Scales, 2nd Edition (PDMS-2)	11 (6%)	8/9
Early Feeding Skills Assessment (EFS)	11 (6%)	8/9
Newborn Individualized Developmental Care and Assessment Program (NIDCAP)	10 (5%)	10
Infant Positioning Assessment Tool (IPAT)	7 (4%)	11
Neonatal Oral-Motor Assessment Scale (NOMAS)	6 (3%)	12/13/14
Neonatal Eating Outcome Assessment Tool (NEO)	6 (3%)	12/13/14
Newborn Behavioral Observation (NBO)	6 (3%)	12/13/14
Infant Neurological International Battery (INFANIB)	5 (3%)	15/16
Neonatal Intensive Care Unit Network Neurobehavioral Scale (NNNS)	5 (3%)	15/16
TIMP Screen (TIMPS)	4 (2%)	17/18
Hammersmith Infant Neurological Examination (HINE)	4 (2%)	17/18
Ballard	2 (1%)	19/20/21/22/23/24
Infant Driven Feeding Scale (IDFS)	2 (1%)	19/20/21/22/23/24
Hawaii Early Learning Profile (HELP)	2 (1%)	19/20/21/22/23/24
Gesell Developmental Observation	2 (1%)	19/20/21/22/23/24
Assessment of Preterm Infant Behavior (APIB)	2 (1%)	19/20/21/22/23/24
Fiorentino reflex testing	1 (1%)	25
Bayley Infant Neurodevelopmental Scale (BINS)	1 (1%)	25
Canadian Infant and Toddler Screening Network (CCITSN)	1 (1%)	21
Latch, Audible swallowing, Type of nipple, Comfort, and Hold (LATCH)	1 (1%)	25
Flexible Endoscopic Evaluation of Swallowing (FEES)	1 (1%)	25
Modified Barium Swallow (MBS)	1 (1%)	25
Bedside swallow evaluation	1 (1%)	25
Sleep readiness	1 (1%)	25
Rosetti Infant-toddler language scale	2 (1%)	19/20/21/22/23/24
Lacey Assessment of Preterm Infants (LAPI)	2 (1%)	19/20/21/22/23/24
Test of Sensory Integration and Functioning in Infants (TSIFI)	1 (1%)	25/26/27/28/29/30/31/32/33/34/35/36
Erhardt Developmental Prehension Assessment	1 (1%)	25/26/27/28/29/30/31/32/33/34/35/36
Erhardt Vision Assessment	1 (1%)	25/26/27/28/29/30/31/32/33/34/35/36
Safe Sleep Assessment Tool (SSAT)	1 (1%)	25/26/27/28/29/30/31/32/33/34/35/36
Neurodevelopmental treatment (NDT)	1 (1%)	25/26/27/28/29/30/31/32/33/34/35/36
Posture and Fine Motor Assessment for Infants (PFMAI)	1 (1%)	25/26/27/28/29/30/31/32/33/34/35/36
Premie-Neuro Exam (PNE)	1 (1%)	25/26/27/28/29/30/31/32/33/34/35/36
Swallowing Assessment of Feeding Evaluation (SAFE)	1 (1%)	25/26/27/28/29/30/31/32/33/34/35/36
The Behavioral Signs of Respiratory Instability (BSRI) Scale	1 (1%)	25/26/27/28/29/30/31/32/33/34/35/36
Neonatal Pain, Agitation, and Sedation Scale (N-PASS)	1 (1%)	25/26/27/28/29/30/31/32/33/34/35/36
Developmental Assessment of Young Children (DAYC)	1 (1%)	25/26/27/28/29/30/31/32/33/34/35/36
Sensory Profile	1 (1%)	25/26/27/28/29/30/31/32/33/34/35/36
Feeding and Nutrition Assessment (FNAST)	1 (1%)	26

a Therapists wrote in freehand the assessments that they use with the NICU population. Of note, these have not been screened to determine their appropriateness for this population nor if they are formalized assessments. In addition, some therapists also wrote in tools that they used in follow-up clinics. Due to some of these issues, we have listed the top 15 tools to include TIMP, GMA, Bayley, NBAS, AIMS, Hammersmith, Morgan, Peabody, EFS, NIDCAP, IPAT, NOMAS, NEO, and NBO.

Productivity standards are common among neonatal therapists, with 415 (65.3%) respondents reporting their existence, while standards ranged from 50%–80% and 11–22 relative value units (RVUs) per workday. Some neonatal therapists reported having to adhere to general hospital standards, while others reported these may be modified or even eliminated for NICU therapists. Free response comments about productivity were summarized with ChatGPT and yielded the following summary: While productivity targets for therapists across different settings generally range from 50%–80%, the unique demands of NICU care require more flexible standards, with many therapists struggling to meet traditional productivity expectations due to the specialized nature of their work.

### 3.4 NICU demographics and characteristics

The majority of neonatal therapists reported working in level III NICUs (760, 59.1%), while 273 (21.2%) worked in level IV NICUs, and 248 (19.3%) reported working in level II NICUs. Although level I NICUs are not considered intensive care, 6 (0.5%) reported working in level I NICUs. The number of NICU beds served in the therapists' hospital ranged from 2–173 with a median of 29.5 beds (IQR 16.0–48.0). The number of therapy FTEs ranged from 0–40, with median 2.0 and IQR 1.0–3.7. The calculated variable FTE per bed was based on the number of beds reported for a given NICU, with a median of 0.0593 FTE per bed (IQR 0.0399–0.0929). Taking the inverse of this, we observe a median of 1 therapy FTE per 17 NICU beds (IQR 1 FTE per 10.8–25.0 beds, range 1 FTE per 10–200 beds).

Neonatal therapy survey respondents reported working with a median of 76.0% of infants in their respective NICUs (IQR 65.0–90.0, range 1%–100%). In terms of how therapy orders are generated, 294 (46%) respondents reported having standing orders, 191 (29.9%) reported getting orders on a case by case basis, 53 (4%) indicated it was based on a therapy screen, 54 (4.1%) reported it was based on a medical screen, and 47 (3.6%) indicated another way of receiving orders (which included automatic orders with specific criteria, screening charts, etc.).

### 3.5 Relationships with productivity standards

Therapists who reported having a productivity standard were more likely to work in a higher level NICU (*β* = 0.84, SE = 0.145, *p* < 0.001), have more NICU beds (*β* = 0.021, SE = 0.004, *p* < 0.001), but have a smaller percentage of their time spent working in the NICU (*β* = −0.217, SE = 0.056, *p* < 0.001), and not be likely to report directly to the NICU (*β* = −0.22, SE = 0.19, *p* < 0.001). There was no relationship between having a productivity standard and therapy discipline (*β* = 0.041, SE = 0.100, *p* = 0.682).

### 3.6 Relationships with number of FTEs

Higher number of reported NICU therapy FTEs were related to higher NICU acuity level (*β* = 2.23, SE = 0.197, *p* < 0.001) and higher number of NICU beds (*β* = 2.497, SE = 0.285, *p* < 0.001). Higher number of beds per FTE was not related to higher percentage of infants served in the NICU (*β* = 14.931, SE = 8.3, *p* = 0.073). Higher number of beds per FTE was related to being a CNT (*β* = 1.066, SE = 0.478, *p* = 0.026).

### 3.7 Relationships with how NICU therapy orders are made

Provision of standing orders for NICU therapy was related to a higher percentage of infants served in the NICU (*β* = −1.109, S = 0.393, *p* < 0.001) and to whether there is a productivity standard *β* = −0.467, SE = 0.139, *p* < 0.001). There were no relationships observed between having standing orders and NICU level, number of beds, whether the therapist reports directly to the NICU, or number of FTEs in the NICU.

### 3.8 Relationships with percentage of infants in the NICU served by therapy

Higher percentage of infants served was related to higher NICU acuity level (*β* = 4.358, SE = 1.517, *p* = 0.004), higher number of NICU beds (*β* = 0.058, SE = 0.029, *p* = 0.047), having a productivity standard (*β* = 11.47, SE = 1.9, *p* < 0.001), and higher number of FTEs (*β* = 1.0, SE = 0.239, *p* < 0.001). Whether therapists report directly to the NICU was not related to the percentage of infants in the NICU served by therapy. Having the status of CNT was related to a higher percentage of infants served in the NICU (*β* = −3.2, SE = 0.589, *p* < 0.001).

## 4 Discussion

The key findings of this survey research are that neonatal therapists engage in multiple, varied experiences and education to perform in their job. There is a growing number of neonatal therapists, but the make-up of therapy teams in different NICUs remains variable. Similarly, the referral process, the number of FTEs, and whether there is a productivity standard varies from hospital to hospital. This updated practice analysis portrays the changing landscape of a growing field. Further, it identifies how different components of neonatal therapy practice can impact the infants and families served.

The number of neonatal therapists and CNTs appears to be growing, as the number of neonatal therapy respondents in the 2019–2020 survey more than doubled from the previous practice analysis of 2015–2016 (11). The largest representation being from OTs is consistent, but it appears that SLPs have closed the gap with PTs, and both are equally represented after OTs in the population of neonatal therapists in 2020. While the practice analysis demonstrates the variability which exists in areas of neonatal therapy, it also serves to identify common areas of practice and knowledge and reminds us that as clinicians what unites us is stronger than what separates us by discipline, geography, unit setting or level. The current findings are consistent with the previous practice analysis in 2015–2016 (10), and ensure that the exam content for certification remains current, relevant, and aligned with real-world practice, as it is updated with each practice analysis.

 The practice analysis affirms that neonatal therapy is an advanced area of practice, as recognized by AOTA, APTA, ASHA and international therapy organizations; and supports the need for experienced neonatal therapists in level II, III & IV NICUs as advocated by major physician and nursing organizations in the US and Canada (5, 14). In terms of clinical and practical implications for the future of neonatal therapy, the practice analysis delineates expectations in terms of hospital roles and reporting structure for neonatal therapists as well as productivity standards. While some neonatal therapists did not report directly to the NICU from an administrative perspective, this does not change the interdisciplinary nature of their NICU work, or their collaborative participation in NICU care from admission to discharge. However, identifying neonatal therapist reporting patterns is still relevant, because they may have an impact on the understanding and use of performance indicators such as productivity.

Our findings demonstrate the growing presence, roles, and relevance of therapists in the NICU. For the first time, to our knowledge, here we report on neonatal therapist involvement in developmental care committees, developmental rounds, and other leadership tasks associated with NICU care. Such descriptive information will enable comparisons in future practice analyses to observe if practice continues to change and evolve. Although there is variability in practice, 24% of respondents identified the existence of standing orders in their NICU. The use of automatic orders has been studied in emergency situations such as with the use of naloxone after opioid exposure (15). In regard to therapeutic programming, standing orders for cardiac rehabilitation have been shown to increase utilization (16). It seems that the use of automatic orders for services such as therapy in the NICU has not been investigated in depth before, but could increase access to timely services as well as remove bias from the referral process. Utilization of such standing orders was related to a higher percentage of infants served in the NICU in the current study, supporting the notion of improved access to care. The use of standing orders was also related to a productivity standard. Since both standing orders and productivity standards are adopted practices in a NICU, it remains unclear if these decisions go hand in hand or if one may impact the other. However, having standing orders for certain high-risk populations decreases the time that therapists must work on communication and screening to garner appropriate referrals, thus enabling more time to dedicate to impactful interventions.

With growing numbers of neonatal therapists and new guidelines for experienced therapy teams in the NICU, we observed a median of 3 neonatal therapy FTEs per unit equating to a median of 1 FTE per 17 beds, with higher numbers reported among hospitals with high acuity levels. This is consistent with the 2015–2016 practice analysis, where 32% of respondents had 1 FTE per 0–10 beds and 33% had 1 FTE for 11–20 beds (10). Further, more NICU beds were related to a larger number of FTEs, which ensures that there is appropriate coverage for infants in the NICU and aligns with other publications on this issue that have made recommendations for minimum staffing calculations (6).

We identified that therapists serve an average of 72% of infants in the NICU. This demonstrates some increase from previous reports in 2015–2016, where only 64% of respondents indicated that therapy was provided to more than 50% of infants in their NICU (10). Higher acuity NICUs, NICUs with more beds and FTEs, as well as those with more CNTs were all related to a higher percentage of infants being served in the NICU. In addition, respondents who were CNTs reported more FTEs per NICU beds. This could demonstrate the strong role of increased knowledge, experience, and advocacy that CNTs bring to the NICU to increase therapy presence and access to infants and families. Interestingly, having a productivity standard was related to a higher percentage of infants served. While this could be a natural relationship between productivity standards and more expansive therapy programs, it could also be evidence that productivity standards could have a positive impact on promoting more infants to be served. Surprisingly, whether or not therapists report directly to the NICU was not related to the percentage of infants in the NICU served by therapy, meaning that effective programming may be driven by other factors besides therapy team reporting hierarchies.

This survey-based research had limitations. We were not able to get full representation among all neonatal therapists, and there is likely inherent bias in those willing to answer a survey on current practice. Despite the validation of the survey, questions can be interpreted in different ways, impacting consistent answers of each cue. Some data had a qualitative component, making it challenging to pull together into a collective whole for ease of interpretation. Some therapists have worked in more than one setting, or practice is changing within their current setting, making it challenging to answer some questions definitively. For instance, since many neonatal therapists also have clinical responsibilities in follow-up clinics, the assessment data reported included tools that may be appropriate in a follow-up setting for NICU graduates rather than during the initial NICU stay, such as use of the Bayley III. In addition, there can be different interpretations of NICU level, especially when comparing US classifications to those used in Europe, which could have affected clarity in reporting NICU acuity level. Not all respondents completed every question, so there was missing data. It is unclear if this was due to survey abandonment or question fatigue, or if respondents decided not to complete certain questions. There was no verification that a therapist only took the survey once, and due to the length of time the survey was open, it is possible that therapists completed another survey not realizing they already had completed one. The survey time period extended into 2020, during a time when life was different than any other time due to stay-at-home orders and changes in NICU policies with the onset of the COVID-19 pandemic, which could have impacted how therapists responded. We used all available data, so there may be multiple representations in the survey from therapists at the same hospital.

## 5 Conclusion

This manuscript provides an analysis of neonatal therapy practice in 2019/2020 and recognizes changes in practice since a previous survey was completed in 2015–2016. The number of neonatal therapists has increased significantly, and the percentage of infants served in the NICU has grown, particularly for higher acuity NICUs, NICUs with more beds and FTEs, as well as those with more CNTs. While all three rehabilitation professions are involved in neonatal therapy, team composition varied across hospitals. Respondents identified areas of treatment focus, standardized assessments used, and the environment of practice provision, including orders for intervention and productivity standards. In addition, neonatal therapists reported increasingly larger roles in medical and developmental rounds and leadership roles, including developmental care committees and staff education.

## Data Availability

The raw data supporting the conclusions of this article will be made available by the authors, upon appropriate request and agreements in place.
