# Supplementary material for: An evolving role in the NICU: a study of the tasks, knowledge, skills, and practice demographics of the neonatal therapist
Source: Front Pediatr. 2025 Nov 26;13:1677432. doi: 10.3389/fped.2025.1677432 (PMC12689580; doi:10.3389/fped.2025.1677432)
Supplement: Supplementary file 1 [file Datasheet1.pdf]

Neonatal Therapy Practice Analysis Survey

Please complete the survey below.

Thank you!

Dr. Roberta Pineda at Washington University in St. Louis is conducting a research study that aims to identify the make-up of neonatal therapists.

Any health care professional with knowledge about the make-up of therapists in their hospital can answer 5 questions to aid our understanding.

Subsequent questions are directed toward neonatal therapists who have or are currently working in a NICU with the purpose being to identify and describe current neonatal therapists and their settings worldwide, and determine what key knowledge is necessary for those who wish to practice neonatal therapy.

If you agree to participate, the first 5 questions should take less than 2 minutes. If you are a neonatal therapist and agree to complete the entire survey, it could take up to 20 minutes.

At the end of the survey, you may choose to have your personal data included in a database to be held by the NTNCB for further research related to neonatal therapy national certification. All personal information will be separated from survey data prior to analysis in order to maintain confidentiality of respondents.

Your participation is voluntary. You may choose not to take part at all. If you decide to be in this study, you may stop participating at any time. Any data that was collected as part of your participation in the study will remain as part of the study records and cannot be removed.

We encourage you to ask questions. If you have any questions about the research study itself, please contact: Roberta Pineda 314-266-1304. If you have questions, concerns, or complaints about your rights as a research participant, please contact the Human Research Protection Office at 660 South Euclid Avenue, Campus Box 8089, St. Louis, MO 63110, 1 (800) 438-0445 or email hrpo@wustl.edu. General information about being a research participant can be found on the Human Research Protection Office web site, http://hrpo.wustl.edu/. To offer input about your experiences as a research participant or to speak to someone other than the research staff, call the Human Research Protection Office at the number above.

Thank you very much for considering participation in this research study.

What is the name of the primary hospital in which you provide services in the NICU? ________________________________

What is the highest level of NICU bed at your primary hospital?

- ◯ Level I
- ◯ Level II
- ◯ Level III
- ◯ Level IV
- ◯ Dont Know

What are the total number of NICU beds at your primary hospital? ________________________________

What are the total number of full-time equivalent OT/PT/SLP positions designated for the NICU at your primary hospital? ________________________________
((You may use fractions))

Which best describes the make-up of therapists in the NICU at your primary hospital?

◯ Neonatal therapy largely driven by OT ◯ Neonatal therapy largely driven by PT ◯ Neonatal therapy largely driven by SLP ◯ Neonatal therapy largely driven by OT and PT ◯ Neonatal therapy largely driven by OT and SLP ◯ Neonatal therapy largely driven by PT and SLP ◯ Multi-disciplinary team of all three disciplines but some may work more predominantly with infants in the NICU ◯ Multi-disciplinary team of all three disciplines-all three work with infants in the NICU equally ◯ Other

What is your discipline?

◯ Occupational Therapist ◯ Physical Therapist ◯ Speech-Language Pathologist ◯ Neonatologist ◯ Neonatal Nurse ◯ Other

Would you like to continue taking the practice analysis survey?

◯ Yes ◯ No

How many years of experience do you have as a credentialed professional? ______________________________

What area(s) of practice are you currently working in?

What activities have you done to hone your skills in the NICU? (i.e. observation, courses, mentoring, etc.) ((Please list activities and how many hours you have spent on each))

What barriers exist to you practicing in the NICU?

What would be helpful to you as a clinician who wants to enter practice in the NICU?

Do you wish to provide your email?

◯ Yes ◯ No

Please state the best email address that we can reach you at in the future: ______________________________

Do you have any other questions, comments, or concerns?

◯ Yes ◯ No

Please Specify/Comments:

In total, how many hours per week do you currently work?

____
((Please include ALL settings in which you are employed as a therapist))

On average, how many hours per week do you work in the NICU?

____
((Please include ALL hospitals that you work in))

What are the total number of hours of practice you have in the NICU?

____

Please estimate this based on an average amount of hours worked in the NICU during a typical work week (not based on billable units). Then multiply by the number of weeks you have been in that position.

For example:

A therapist working 40 hours per week or full time in the NICU for one year has 2080 total hours in the NICU.
(40 hours x 52 weeks)

A therapist working approximately 20 hours per week in the NICU for 3 years has 3120 total hours in the NICU.
[(20 hours x 52 weeks) x 3 years]

A therapist working an average of 10 hours per week for 8 years has 4160 total hours in the NICU.
[(10 hours x 52 weeks) x 8 years]

How many hours of NICU related education have you had in the past 3 years?

____

Have you received at least 40 hours of mentoring in the NICU?

◯ Yes ◯ No

How do you keep up with current practice in the NICU?
(Check all that apply)

☐ Read journal articles or books ☐ Attend hospital based education ☐ Attend education outside the hospital ☐ Collaborate with others with expertise ☐ Other

Please Specify:

What is your current position and in what capacity do you work? (please indicate how this does or does not relate to neonatal therapy)

What percentage of time do you spend in the NICU doing direct patient care, evaluation, treatment, parent teaching, and/or staff collaboration during your typical work hours?

◯ All of my time ◯ > 75% ◯ 50-75% ◯ 25-49% ◯ 10-24% ◯ < 10% or inconsistent part of my employment ◯ I am primarily asked to consult in the NICU

When you were working in the NICU, what percentage of time did you spend in the NICU doing direct patient care, evaluation, treatment, parent teaching, and/or staff collaboration during your typical work hours?

◯ All of my time ◯ > 75% ◯ 50-75% ◯ 25-49% ◯ 10-24% ◯ < 10% or inconsistent part of my employment ◯ I am primarily asked to consult in the NICU

If some of your time at your primary hospital is spent on non-NICU related services, what area(s) are the other parts of your time dedicated to?
(Check all that apply)

☐ Administration ☐ Adult inpatient ☐ Adult outpatient ☐ Mother-baby ☐ NICU follow-up clinic ☐ Pediatric acute inpatient, specifically pediatric or cardiac intensive care unit only ☐ Pediatric acute inpatient, areas outside of pediatric and cardiac intensive care unit ☐ Pediatric inpatient rehabilitation ☐ Pediatric outpatient ☐ Developmental daycare ☐ Schools ☐ Skilled nursing ☐ Other ☐ N/A (All of my time is spent on NICU related services)

Please Specify:

If some of your time at your primary hospital was spent on non-NICU related services, what area(s) were the other parts of your time dedicated to?
(Check all that apply)

☐ Administration ☐ Adult inpatient ☐ Adult outpatient ☐ Mother-baby ☐ NICU follow-up clinic ☐ Pediatric acute inpatient, specifically pediatric or cardiac intensive care unit only ☐ Pediatric acute inpatient, areas outside of pediatric and cardiac intensive care unit ☐ Pediatric inpatient rehabilitation ☐ Pediatric outpatient ☐ Developmental daycare ☐ Schools ☐ Skilled nursing ☐ Other ☐ N/A (All of my time was spent on NICU related services)

Please Specify:

Please Specify/Comments:

What percentage of the infants hospitalized at your primary hospital receive therapy services?

0% 50% 100%

(Place a mark on the scale above)

What percentage of the infants hospitalized at your primary hospital received therapy services?

0% 50% 100%

(Place a mark on the scale above)

What are most of the therapy referrals requesting of your services? (i.e. feeding, oral-motor evaluations, splinting, etc.)

How are orders received by the therapy team at your primary hospital?

◯ Standing orders ◯ Orders written on a case by case basis ◯ Orders based on screening of infant done by the therapy team ◯ Orders based on screening done of medical risk factors ◯ Other

Please Specify/Comments:

Who do you report to?
(Check all that apply)

☐ Adult Rehab ☐ Peds Rehab ☐ NICU directly ☐ Other

Please Specify/Comments:

What services do you perform in the NICU at your primary hospital?
(Check all that apply)

☐ Use developmental care principles ☐ Use a habilitative/preventative model ☐ Use a rehabilitative (problem-based) model ☐ Attend medical or developmental rounds ☐ Contribute to medical or developmental rounds ☐ Educate parents ☐ Educate nursing ☐ Educate physicians ☐ Conduct feeding related treatment ☐ Conduct feeding studies (MBS or FEES) ☐ Conduct positioning treatment ☐ Do splinting ☐ Do chest physiotherapy ☐ Conduct movement related treatments ☐ Conduct sensory based treatments ☐ Co-treat with other therapists ☐ Engage in discharge planning/make recommendations for discharge ☐ Make referrals ☐ Communicate with therapists in the community to bridge services from NICU to home ☐ Conduct standardized assessments for clinical purposes ☐ Conduct standardized assessments for research ☐ Engage in research activities (on someone else's study) ☐ Engage in research activities (on my own study) ☐ Contribute to the development of a bedside care plan for patients and families ☐ Other

Please Specify:

Please list all of the standardized assessments you use for clinical purposes:

Please list all of the standardized assessments you use for research:

In your NICU, are there developmental rounds?

◯ Yes ◯ No

Are therapists in the unit active participants?

◯ Yes ◯ No

Why not?

Do neonatal therapists have a leadership role?

◯ Yes ◯ No

Why not?

Did neonatal therapists have a leadership role?

◯ Yes ◯ No

In your NICU, is there developmental care committee?

◯ Yes ◯ No

Are therapists in the unit active participants?

◯ Yes ◯ No

Why not?

Do neonatal therapists have a leadership role?

◯ Yes ◯ No

Do neonatal therapists in your unit participate in daily medical rounds?

◯ Yes ◯ No

How often?

◯ Daily ◯ A few times per week ◯ Weekly ◯ Twice per month ◯ Monthly ◯ Other

Please Specify:

Do neonatal therapists in your unit participate in clinical leadership meetings?

◯ Yes ◯ No

How often?

◯ Daily ◯ A few times per week ◯ Weekly ◯ Twice per month ◯ Monthly ◯ Other

Please Specify:

Did neonatal therapists in your unit participate in clinical leadership meetings?

◯ Yes ◯ No

How often?

◯ Daily ◯ A few times per week ◯ Weekly ◯ Twice per month ◯ Monthly ◯ Other

Please Specify:

Do you have a productivity standard at your primary hospital? (i.e. certain number of units/babies you should see each day)

◯ Yes ◯ No

Please Specify:

Do you currently work in the NICU at any other hospitals? (other than your primary hospital)

◯ Yes ◯ No

Please Specify:
(Please include: Name, City, State, Country)

In the past, were there other hospitals that you worked for in the NICU?

◯ Yes ◯ No

Please Specify:
(Please include: Name, City, State, Country)

Do you wish to provide your email?

◯ Yes ◯ No

Please state the best email address that we can reach you at in the future: ______________________________

What is your current status?

◯ Certified Neonatal Therapist (CNT) ◯ In process of becoming a CNT ◯ Not a CNT but working toward requirements ◯ Not a CNT but interested in becoming a CNT ◯ Not a CNT and unsure if I will become a CNT ◯ Not a CNT and not interested in becoming a CNT
